# 1-[((*E*)-{2-[(2-Nitro­benz­yl)(2-{[(*E*)-(2-oxidonaphthalen-1-yl)methyl­idene]aza­nium­yl}eth­yl)amino]­eth­yl}aza­niumyl­idene)meth­yl]naphthalen-2-olate monohydrate

**DOI:** 10.1107/S1600536811047465

**Published:** 2011-11-16

**Authors:** Saeid Menati, Hadi Amiri Rudbari, Reza Azadbakht, Giuseppe Bruno

**Affiliations:** a Department of Chemistry, Islamic Azad University, Khorramabad Branch, Khorramabad, Iran; b Dipartimento di Chimica Inorganica, Vill. S. Agata, Salita Sperone 31, Universita di Messina 98166 Messina, Italy; c Department of Chemistry, Payame Noor University, Hamedan, Iran

## Abstract

The title Schiff base compound, C_33_H_30_N_4_O_4_·H_2_O, adopts an *E* configuration with respect to each C=N double bond. In the mol­ecule, there are naphthoxide anions and the protonated imino N atoms. Intra­molecular N—H⋯O hydrogen bonds lead to the formation of approximately planar (maximum deviation 0.029 Å for H atom) six-membered rings.. In the crystal, mol­ecules are linked by O—H⋯O and N—H⋯O hydrogen bonds as well as C—H⋯O contacts, leading to the formation of a three-dimensional network.

## Related literature

For related structures, see: Keypour *et al.* (2008 ▶); Zeng *et al.* (1999 ▶); McKee *et al.* (2006 ▶). For Schiff base derivatives incorporating a fluorescent moiety as tools for optical sensing of metal ions, see: Aza­dbakht *et al.* (2011 ▶). 
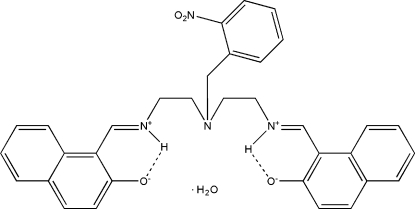


## Experimental

### 

#### Crystal data

C_33_H_30_N_4_O_4_·H_2_O
*M* *_r_* = 564.63
Monoclinic, 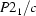

*a* = 13.8808 (6) Å
*b* = 14.8951 (8) Å
*c* = 14.7517 (8) Å
β = 111.754 (2)°
*V* = 2832.8 (2) Å^3^
*Z* = 4
Mo *K*α radiation
μ = 0.09 mm^−1^
*T* = 296 K
0.5 × 0.3 × 0.2 mm

#### Data collection

Bruker APEXII CCD diffractometer
Absorption correction: multi-scan (*SADABS*; Bruker, 2008) ▶ *T*_min_ = 0.671, *T*_max_ = 0.745
27583 measured reflections
5261 independent reflections
3757 reflections with *I* > 2σ(*I*)
*R*_int_ = 0.027

#### Refinement

*R*[*F* ^2^ > 2σ(*F* ^2^)] = 0.058
*wR*(*F* ^2^) = 0.195
*S* = 1.04
5261 reflections
395 parameters
2 restraints
H atoms treated by a mixture of independent and constrained refinement
Δρ_max_ = 0.53 e Å^−3^
Δρ_min_ = −0.35 e Å^−3^

### 

Data collection: *APEX2* (Bruker, 2007 ▶); cell refinement: *SAINT* (Bruker, 2007 ▶); data reduction: *SAINT*; program(s) used to solve structure: *SHELXS97* (Sheldrick, 2008 ▶); program(s) used to refine structure: *SHELXL97* (Sheldrick, 2008 ▶); molecular graphics: *XP* in *SHELXTL* (Sheldrick, 2008 ▶); software used to prepare material for publication: *SHELXTL*.

## Supplementary Material

Crystal structure: contains datablock(s) I, global. DOI: 10.1107/S1600536811047465/qm2040sup1.cif

Structure factors: contains datablock(s) I. DOI: 10.1107/S1600536811047465/qm2040Isup2.hkl

Supplementary material file. DOI: 10.1107/S1600536811047465/qm2040Isup3.cml

Additional supplementary materials:  crystallographic information; 3D view; checkCIF report

## Figures and Tables

**Table 1:** Hydrogen-bond geometry (Å, °)

*D*—H⋯*A*	*D*—H	H⋯*A*	*D*⋯*A*	*D*—H⋯*A*
N2—H2⋯O2	0.79 (3)	1.99 (3)	2.596 (3)	133 (3)
N3—H3⋯O1	0.84 (3)	1.88 (3)	2.567 (3)	138 (3)
O5—H5*D*⋯O1^i^	0.95 (4)	1.82 (4)	2.747 (3)	164 (5)
N2—H2⋯O4^ii^	0.79 (3)	2.60 (3)	3.183 (4)	131 (2)
O5—H5*C*⋯O2^iii^	0.94 (3)	1.91 (3)	2.839 (3)	170 (3)
C1—H1*A*⋯O5^iv^	0.97	2.53	3.409 (4)	151
C2—H2*A*⋯O5^iv^	0.97	2.55	3.359 (4)	140

Symmetry codes: (i) 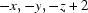; (ii) 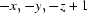; (iii) 
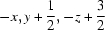
; (iv) 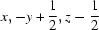.

**Fig. 1.**
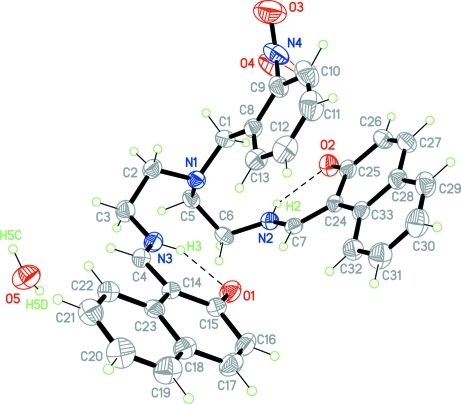
Perspective view of the title molecule with numbering of the atoms. Non H-atoms represented as displacement ellipsoids are plotted at the 30% probability level, while H atoms are shown as small spheres of arbitrary radius.

**Fig. 2.**
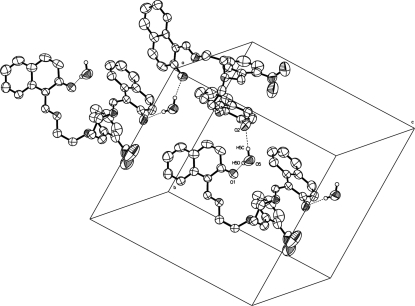
A stereoview of part of the crystal packing, showing the intermolecular hydrogen bonds. For clarity, H atoms have been omitted for clarity except hydrogen of water. Hydrogen bonds are denoted by dashed lines.

## supplementary crystallographic information

### Comment

Schiff bases (imines) are known to be good ligands for metal ions. A number of the Schiff bases have antitumor properties, antioxidative activities, attractive electronic and photophysical properties. In addition, Schiff base derivatives incorporating a fluorescent moiety are appealing tools for optical sensing of metal ions (Azadbakht *et al.* 2011). The title compound, (I), crystallizes in the Monoclinic space group P2(1)/c. Figure 1 shows the *ORTEP* representation of the molecule with thermal ellipsoids at the 30% probability level. In the compound, there are π-π interactions between the naphthalene moiety and the nitrobenzene moiety with the distance of 3.516 (3) Å. The structural features of I (The bond lengths) provides evidence for the existence of a naphthoxide anions and the protonated imino N atoms in the crystal structure of I that stabilize by intramolecular hydrogen bond between the naphtholate oxygen and the iminium nitrogen (O-···H—N+). This molecular conformation is determined by the formation of pairs of intramolecular N±–H···O– (1.888 (3) Å) hydrogen bonds. These interactions lead to the formation of six- membered rings (see scheme). Details of the hydrogen-bonding geometry are given in Table 1. Each of these hydrogen-bonded rings adopts a nearly planar conformation. In the six-membered rings, the maximum deviation from the mean planes is 0.029 Å  for H (N±–H). The crystal packing in compound (1) is stabilized by O—H···O– interactions (Fig. 2 and Table 1).

### Experimental

A solution of NaOH (3 mmol) in methanol (10 *cm*3) was added to a suspension of N1-(2-nitrobenzyl)-N1-(2-aminoethyl)ethane-1,2- diamine trihydrochloride (0.357 g, 1 mmol) in methanol (10 *cm*3). The mixture was stirred at room temperature for a few minutes then filtered, and the precipitate was washed well with methanol (10 *cm*3). The washings and the filtrate were combined and to this solution was added 2-hydroxy-1-naphthaldehyde (0.344 g, 2 mmol). After refluxing the solution for 8 h on cooling the product was recovered as a yellow powder, which was recrystallized from methanol.

### Refinement

Several H atoms were located on final difference map. However, the H atoms were included in the refinement using a riding model with the *X*—H bond geometry and the H isotropic displacement parameter depending on the parent atom*X*.

### Crystal data

**Table:**

C_33_H_30_N_4_O_4_·H_2_O	*F*(000) = 1192
*M**_r_* = 564.63	*D*_x_ = 1.324 Mg m^−^^3^
Monoclinic, *P*2_1_/*c*	Mo *K*α radiation, λ = 0.71073 Å
Hall symbol: -P 2ybc	Cell parameters from 7294 reflections
*a* = 13.8808 (6) Å	θ = 2.2–24.5°
*b* = 14.8951 (8) Å	µ = 0.09 mm^−^^1^
*c* = 14.7517 (8) Å	*T* = 296 K
β = 111.754 (2)°	Irregular, yellow
*V* = 2832.8 (2) Å^3^	0.5 × 0.3 × 0.2 mm
*Z* = 4	

### Data collection

**Table:**

Bruker APEXII CCD diffractometer	5261 independent reflections
Radiation source: fine-focus sealed tube	3757 reflections with *I* > 2σ(*I*)
graphite	*R*_int_ = 0.027
φ and ω scans	θ_max_ = 25.5°, θ_min_ = 2.7°
Absorption correction: multi-scan (*SADABS*; Bruker, 2008)	*h* = −16→16
*T*_min_ = 0.671, *T*_max_ = 0.745	*k* = −18→18
27583 measured reflections	*l* = −17→17

### Refinement

**Table:**

Refinement on *F*^2^	Primary atom site location: structure-invariant direct methods
Least-squares matrix: full	Secondary atom site location: difference Fourier map
*R*[*F*^2^ > 2σ(*F*^2^)] = 0.058	Hydrogen site location: inferred from neighbouring sites
*wR*(*F*^2^) = 0.195	H atoms treated by a mixture of independent and constrained refinement
*S* = 1.04	*w* = 1/[σ^2^(*F*_o_^2^) + (0.1034*P*)^2^ + 1.1914*P*] where *P* = (*F*_o_^2^ + 2*F*_c_^2^)/3
5261 reflections	(Δ/σ)_max_ < 0.001
395 parameters	Δρ_max_ = 0.53 e Å^−^^3^
2 restraints	Δρ_min_ = −0.35 e Å^−^^3^

### Special details

**Table:**

Geometry. All s.u.'s (except the s.u. in the dihedral angle between two l.s. planes) are estimated using the full covariance matrix. The cell s.u.'s are taken into account individually in the estimation of s.u.'s in distances, angles and torsion angles; correlations between s.u.'s in cell parameters are only used when they are defined by crystal symmetry. An approximate (isotropic) treatment of cell s.u.'s is used for estimating s.u.'s involving l.s. planes.
Refinement. Refinement of *F*^2^ against ALL reflections. The weighted *R*-factor *wR* and goodness of fit *S* are based on *F*^2^, conventional *R*-factors *R* are based on *F*, with *F* set to zero for negative *F*^2^. The threshold expression of *F*^2^ > 2σ(*F*^2^) is used only for calculating *R*-factors(gt) *etc*. and is not relevant to the choice of reflections for refinement. *R*-factors based on *F*^2^ are statistically about twice as large as those based on *F*, and *R*- factors based on ALL data will be even larger.

### Fractional atomic coordinates and isotropic or equivalent isotropic displacement parameters (Å^2^)

**Table:**

	*x*	*y*	*z*	*U*_iso_*/*U*_eq_	
N2	0.02034 (16)	−0.10330 (14)	0.67594 (18)	0.0489 (5)	
O5	−0.0441 (2)	0.22187 (17)	1.08799 (17)	0.0868 (7)	
N3	0.08388 (19)	0.08971 (16)	0.95583 (16)	0.0575 (6)	
O1	0.15835 (17)	−0.07002 (12)	0.98645 (15)	0.0683 (5)	
O2	0.08995 (15)	−0.12590 (13)	0.53670 (13)	0.0642 (5)	
C7	0.11465 (19)	−0.12276 (15)	0.73426 (18)	0.0457 (5)	
H7	0.1288	−0.1223	0.8010	0.055*	
N1	0.04699 (15)	0.08040 (13)	0.75101 (14)	0.0480 (5)	
C14	0.20514 (19)	0.04119 (15)	1.11073 (18)	0.0459 (6)	
C23	0.26511 (17)	0.06422 (16)	1.21139 (18)	0.0465 (6)	
C30	0.4997 (3)	−0.1957 (2)	0.9093 (3)	0.0802 (9)	
H30	0.5664	−0.2060	0.9542	0.096*	
C31	0.4165 (3)	−0.1976 (2)	0.9386 (2)	0.0766 (9)	
H31	0.4273	−0.2098	1.0035	0.092*	
C32	0.3183 (2)	−0.18192 (19)	0.8738 (2)	0.0615 (7)	
H32	0.2632	−0.1837	0.8953	0.074*	
C33	0.29846 (19)	−0.16309 (15)	0.77513 (18)	0.0488 (6)	
C24	0.19620 (18)	−0.14433 (15)	0.70377 (17)	0.0456 (5)	
C6	−0.05483 (19)	−0.06051 (18)	0.7097 (2)	0.0535 (6)	
H6A	−0.0383	−0.0755	0.7778	0.064*	
H6B	−0.1235	−0.0838	0.6726	0.064*	
C5	−0.05569 (19)	0.04108 (18)	0.69884 (19)	0.0525 (6)	
H5A	−0.0782	0.0563	0.6301	0.063*	
H5B	−0.1051	0.0667	0.7240	0.063*	
C2	0.0439 (2)	0.16542 (17)	0.80026 (19)	0.0580 (7)	
H2A	−0.0060	0.2051	0.7543	0.070*	
H2B	0.1114	0.1939	0.8202	0.070*	
C3	0.0154 (2)	0.15386 (19)	0.8878 (2)	0.0622 (7)	
H3A	0.0200	0.2113	0.9202	0.075*	
H3B	−0.0557	0.1330	0.8674	0.075*	
C4	0.1394 (2)	0.10508 (16)	1.04679 (18)	0.0497 (6)	
H4	0.1357	0.1619	1.0715	0.060*	
C18	0.32210 (19)	−0.00420 (18)	1.2758 (2)	0.0561 (7)	
C19	0.3780 (2)	0.0174 (2)	1.3744 (2)	0.0753 (9)	
H19	0.4146	−0.0277	1.4168	0.090*	
C20	0.3802 (2)	0.1015 (2)	1.4094 (2)	0.0769 (9)	
H20	0.4179	0.1138	1.4748	0.092*	
C29	0.4839 (2)	−0.1787 (2)	0.8144 (3)	0.0717 (8)	
H29	0.5403	−0.1774	0.7948	0.086*	
C28	0.3837 (2)	−0.16316 (17)	0.7452 (2)	0.0554 (6)	
C22	0.2694 (2)	0.15089 (17)	1.25023 (19)	0.0538 (6)	
H22	0.2331	0.1971	1.2095	0.065*	
C21	0.3257 (2)	0.1693 (2)	1.3470 (2)	0.0658 (7)	
H21	0.3273	0.2274	1.3705	0.079*	
C17	0.3211 (2)	−0.09229 (19)	1.2381 (2)	0.0659 (8)	
H17	0.3584	−0.1371	1.2805	0.079*	
C16	0.2690 (2)	−0.11355 (18)	1.1444 (2)	0.0644 (8)	
H16	0.2722	−0.1720	1.1236	0.077*	
C15	0.2082 (2)	−0.04778 (16)	1.0751 (2)	0.0528 (6)	
C1	0.10302 (19)	0.09324 (18)	0.68424 (19)	0.0524 (6)	
H1A	0.0845	0.1513	0.6529	0.063*	
H1B	0.0804	0.0477	0.6337	0.063*	
C8	0.21990 (19)	0.08819 (16)	0.7341 (2)	0.0512 (6)	
C13	0.2687 (2)	0.06145 (19)	0.8299 (2)	0.0611 (7)	
H13	0.2285	0.0470	0.8662	0.073*	
C12	0.3750 (2)	0.0555 (2)	0.8734 (3)	0.0786 (9)	
H12	0.4054	0.0371	0.9381	0.094*	
C11	0.4369 (3)	0.0767 (3)	0.8215 (3)	0.0909 (11)	
H11	0.5087	0.0717	0.8505	0.109*	
C10	0.3916 (3)	0.1051 (3)	0.7276 (3)	0.0870 (11)	
H10	0.4323	0.1216	0.6925	0.104*	
C9	0.2849 (2)	0.10932 (19)	0.6845 (2)	0.0627 (7)	
N4	0.2402 (3)	0.1395 (2)	0.5833 (2)	0.0826 (8)	
O4	0.1694 (3)	0.1045 (3)	0.5282 (2)	0.1449 (15)	
O3	0.2758 (3)	0.2062 (2)	0.5574 (3)	0.1365 (12)	
C25	0.1803 (2)	−0.13542 (16)	0.60255 (19)	0.0515 (6)	
C26	0.2703 (2)	−0.13597 (19)	0.5771 (2)	0.0632 (7)	
H26	0.2623	−0.1281	0.5121	0.076*	
C27	0.3654 (2)	−0.14743 (19)	0.6443 (2)	0.0641 (7)	
H27	0.4219	−0.1451	0.6249	0.077*	
H5C	−0.067 (3)	0.270 (2)	1.044 (3)	0.105 (13)*	
H5D	−0.092 (3)	0.177 (3)	1.053 (3)	0.141 (17)*	
H2	0.007 (2)	−0.1011 (18)	0.619 (2)	0.051 (8)*	
H3	0.091 (2)	0.039 (2)	0.935 (2)	0.074 (10)*	

### Atomic displacement parameters (Å^2^)

**Table:**

	*U*^11^	*U*^22^	*U*^33^	*U*^12^	*U*^13^	*U*^23^
N2	0.0522 (13)	0.0490 (11)	0.0453 (13)	−0.0033 (9)	0.0177 (11)	−0.0029 (10)
O5	0.116 (2)	0.0707 (15)	0.0578 (13)	−0.0088 (14)	0.0138 (13)	0.0015 (11)
N3	0.0777 (16)	0.0483 (12)	0.0494 (13)	0.0107 (11)	0.0271 (12)	0.0032 (10)
O1	0.0960 (15)	0.0449 (10)	0.0657 (13)	0.0019 (9)	0.0321 (11)	−0.0041 (9)
O2	0.0672 (12)	0.0779 (13)	0.0439 (10)	0.0098 (10)	0.0163 (9)	−0.0046 (9)
C7	0.0559 (14)	0.0388 (12)	0.0433 (13)	−0.0043 (10)	0.0194 (11)	0.0003 (9)
N1	0.0486 (11)	0.0496 (11)	0.0466 (11)	0.0005 (9)	0.0185 (9)	0.0004 (9)
C14	0.0519 (13)	0.0388 (12)	0.0545 (14)	0.0009 (10)	0.0284 (12)	0.0061 (10)
C23	0.0407 (12)	0.0496 (13)	0.0552 (14)	−0.0022 (10)	0.0245 (11)	0.0057 (11)
C30	0.0637 (19)	0.087 (2)	0.074 (2)	0.0162 (16)	0.0072 (17)	−0.0016 (17)
C31	0.080 (2)	0.084 (2)	0.0556 (17)	0.0185 (17)	0.0130 (16)	0.0047 (15)
C32	0.0633 (17)	0.0674 (17)	0.0532 (16)	0.0104 (13)	0.0206 (13)	0.0040 (13)
C33	0.0572 (15)	0.0383 (12)	0.0517 (14)	0.0018 (10)	0.0210 (12)	−0.0032 (10)
C24	0.0530 (14)	0.0382 (12)	0.0470 (13)	0.0011 (10)	0.0203 (11)	−0.0026 (10)
C6	0.0445 (13)	0.0646 (15)	0.0563 (15)	−0.0067 (11)	0.0244 (12)	−0.0007 (12)
C5	0.0435 (13)	0.0660 (16)	0.0522 (14)	0.0095 (11)	0.0225 (11)	0.0074 (12)
C2	0.0747 (18)	0.0478 (14)	0.0509 (15)	0.0078 (12)	0.0226 (13)	0.0047 (11)
C3	0.0715 (18)	0.0619 (16)	0.0516 (15)	0.0152 (13)	0.0212 (13)	0.0034 (12)
C4	0.0602 (15)	0.0432 (13)	0.0508 (15)	0.0011 (11)	0.0266 (12)	−0.0006 (11)
C18	0.0402 (13)	0.0569 (15)	0.0690 (18)	0.0022 (11)	0.0176 (12)	0.0100 (13)
C19	0.0534 (16)	0.087 (2)	0.070 (2)	0.0121 (15)	0.0056 (14)	0.0173 (17)
C20	0.0633 (18)	0.092 (2)	0.0613 (19)	0.0007 (16)	0.0066 (15)	−0.0062 (17)
C29	0.0544 (16)	0.0715 (19)	0.090 (2)	0.0035 (14)	0.0273 (16)	−0.0062 (17)
C28	0.0564 (15)	0.0457 (13)	0.0652 (17)	−0.0014 (11)	0.0239 (13)	−0.0068 (12)
C22	0.0587 (15)	0.0512 (14)	0.0550 (16)	−0.0014 (11)	0.0250 (13)	0.0026 (11)
C21	0.0641 (17)	0.0697 (18)	0.0621 (18)	−0.0049 (14)	0.0217 (14)	−0.0087 (14)
C17	0.0505 (15)	0.0568 (16)	0.084 (2)	0.0100 (12)	0.0178 (15)	0.0193 (15)
C16	0.0628 (17)	0.0395 (13)	0.091 (2)	0.0042 (12)	0.0288 (16)	0.0040 (13)
C15	0.0587 (15)	0.0437 (13)	0.0616 (17)	−0.0016 (11)	0.0291 (13)	0.0023 (11)
C1	0.0516 (14)	0.0580 (14)	0.0502 (14)	−0.0030 (11)	0.0218 (12)	0.0050 (11)
C8	0.0508 (14)	0.0430 (13)	0.0623 (16)	−0.0042 (10)	0.0239 (12)	−0.0054 (11)
C13	0.0528 (15)	0.0600 (16)	0.0649 (18)	−0.0040 (12)	0.0155 (13)	−0.0007 (13)
C12	0.0623 (19)	0.0710 (19)	0.084 (2)	−0.0035 (15)	0.0057 (17)	−0.0065 (16)
C11	0.0498 (18)	0.095 (3)	0.119 (3)	−0.0051 (17)	0.021 (2)	−0.020 (2)
C10	0.0569 (19)	0.105 (3)	0.108 (3)	−0.0128 (18)	0.041 (2)	−0.020 (2)
C9	0.0611 (17)	0.0603 (16)	0.0742 (19)	−0.0082 (13)	0.0337 (15)	−0.0151 (14)
N4	0.090 (2)	0.101 (2)	0.0743 (19)	−0.0289 (17)	0.0517 (18)	−0.0176 (16)
O4	0.104 (2)	0.246 (4)	0.083 (2)	−0.050 (3)	0.0329 (17)	0.015 (2)
O3	0.184 (3)	0.129 (3)	0.128 (3)	−0.024 (2)	0.094 (2)	0.011 (2)
C25	0.0621 (16)	0.0450 (13)	0.0494 (14)	0.0034 (11)	0.0230 (13)	−0.0054 (11)
C26	0.0756 (19)	0.0688 (17)	0.0542 (16)	0.0034 (14)	0.0343 (15)	0.0019 (13)
C27	0.0651 (18)	0.0650 (17)	0.076 (2)	−0.0045 (13)	0.0421 (16)	−0.0051 (14)

### Geometric parameters (Å, °)

**Table:**

N2—C7	1.305 (3)	C3—H3B	0.9700
N2—C6	1.459 (3)	C4—H4	0.9300
N2—H2	0.79 (3)	C18—C19	1.409 (4)
O5—H5C	0.94 (3)	C18—C17	1.423 (4)
O5—H5D	0.95 (4)	C19—C20	1.351 (5)
N3—C4	1.296 (3)	C19—H19	0.9300
N3—C3	1.455 (3)	C20—C21	1.386 (4)
N3—H3	0.84 (3)	C20—H20	0.9300
O1—C15	1.276 (3)	C29—C28	1.407 (4)
O2—C25	1.278 (3)	C29—H29	0.9300
C7—C24	1.401 (3)	C28—C27	1.434 (4)
C7—H7	0.9300	C22—C21	1.377 (4)
N1—C5	1.468 (3)	C22—H22	0.9300
N1—C2	1.468 (3)	C21—H21	0.9300
N1—C1	1.476 (3)	C17—C16	1.339 (4)
C14—C4	1.410 (3)	C17—H17	0.9300
C14—C15	1.432 (3)	C16—C15	1.441 (4)
C14—C23	1.449 (4)	C16—H16	0.9300
C23—C22	1.405 (3)	C1—C8	1.515 (4)
C23—C18	1.418 (3)	C1—H1A	0.9700
C30—C29	1.358 (5)	C1—H1B	0.9700
C30—C31	1.376 (5)	C8—C13	1.381 (4)
C30—H30	0.9300	C8—C9	1.392 (4)
C31—C32	1.364 (4)	C13—C12	1.378 (4)
C31—H31	0.9300	C13—H13	0.9300
C32—C33	1.406 (4)	C12—C11	1.383 (5)
C32—H32	0.9300	C12—H12	0.9300
C33—C28	1.407 (4)	C11—C10	1.359 (5)
C33—C24	1.447 (3)	C11—H11	0.9300
C24—C25	1.432 (3)	C10—C9	1.379 (4)
C6—C5	1.521 (4)	C10—H10	0.9300
C6—H6A	0.9700	C9—N4	1.459 (4)
C6—H6B	0.9700	N4—O4	1.142 (4)
C5—H5A	0.9700	N4—O3	1.232 (4)
C5—H5B	0.9700	C25—C26	1.432 (4)
C2—C3	1.495 (4)	C26—C27	1.336 (4)
C2—H2A	0.9700	C26—H26	0.9300
C2—H2B	0.9700	C27—H27	0.9300
C3—H3A	0.9700		
			
C7—N2—C6	122.8 (2)	C20—C19—C18	122.1 (3)
C7—N2—H2	119 (2)	C20—C19—H19	119.0
C6—N2—H2	116 (2)	C18—C19—H19	119.0
H5C—O5—H5D	100 (4)	C19—C20—C21	119.5 (3)
C4—N3—C3	125.7 (2)	C19—C20—H20	120.2
C4—N3—H3	116 (2)	C21—C20—H20	120.2
C3—N3—H3	119 (2)	C30—C29—C28	121.1 (3)
N2—C7—C24	124.9 (2)	C30—C29—H29	119.5
N2—C7—H7	117.6	C28—C29—H29	119.5
C24—C7—H7	117.6	C33—C28—C29	119.6 (3)
C5—N1—C2	113.8 (2)	C33—C28—C27	118.6 (2)
C5—N1—C1	110.56 (19)	C29—C28—C27	121.8 (3)
C2—N1—C1	109.7 (2)	C21—C22—C23	121.8 (3)
C4—C14—C15	118.6 (2)	C21—C22—H22	119.1
C4—C14—C23	120.6 (2)	C23—C22—H22	119.1
C15—C14—C23	120.7 (2)	C22—C21—C20	120.2 (3)
C22—C23—C18	117.3 (2)	C22—C21—H21	119.9
C22—C23—C14	123.8 (2)	C20—C21—H21	119.9
C18—C23—C14	118.9 (2)	C16—C17—C18	123.0 (2)
C29—C30—C31	119.6 (3)	C16—C17—H17	118.5
C29—C30—H30	120.2	C18—C17—H17	118.5
C31—C30—H30	120.2	C17—C16—C15	121.4 (3)
C32—C31—C30	120.9 (3)	C17—C16—H16	119.3
C32—C31—H31	119.5	C15—C16—H16	119.3
C30—C31—H31	119.5	O1—C15—C14	122.5 (2)
C31—C32—C33	121.4 (3)	O1—C15—C16	120.2 (2)
C31—C32—H32	119.3	C14—C15—C16	117.3 (2)
C33—C32—H32	119.3	N1—C1—C8	113.9 (2)
C32—C33—C28	117.4 (2)	N1—C1—H1A	108.8
C32—C33—C24	123.7 (2)	C8—C1—H1A	108.8
C28—C33—C24	119.0 (2)	N1—C1—H1B	108.8
C7—C24—C25	118.8 (2)	C8—C1—H1B	108.8
C7—C24—C33	120.1 (2)	H1A—C1—H1B	107.7
C25—C24—C33	120.5 (2)	C13—C8—C9	115.8 (3)
N2—C6—C5	112.2 (2)	C13—C8—C1	122.8 (2)
N2—C6—H6A	109.2	C9—C8—C1	121.4 (2)
C5—C6—H6A	109.2	C12—C13—C8	122.0 (3)
N2—C6—H6B	109.2	C12—C13—H13	119.0
C5—C6—H6B	109.2	C8—C13—H13	119.0
H6A—C6—H6B	107.9	C13—C12—C11	120.4 (3)
N1—C5—C6	111.9 (2)	C13—C12—H12	119.8
N1—C5—H5A	109.2	C11—C12—H12	119.8
C6—C5—H5A	109.2	C10—C11—C12	119.2 (3)
N1—C5—H5B	109.2	C10—C11—H11	120.4
C6—C5—H5B	109.2	C12—C11—H11	120.4
H5A—C5—H5B	107.9	C11—C10—C9	119.8 (3)
N1—C2—C3	113.2 (2)	C11—C10—H10	120.1
N1—C2—H2A	108.9	C9—C10—H10	120.1
C3—C2—H2A	108.9	C10—C9—C8	122.8 (3)
N1—C2—H2B	108.9	C10—C9—N4	117.6 (3)
C3—C2—H2B	108.9	C8—C9—N4	119.6 (3)
H2A—C2—H2B	107.8	O4—N4—O3	119.1 (4)
N3—C3—C2	110.9 (2)	O4—N4—C9	121.0 (3)
N3—C3—H3A	109.5	O3—N4—C9	119.8 (3)
C2—C3—H3A	109.5	O2—C25—C26	120.5 (2)
N3—C3—H3B	109.5	O2—C25—C24	122.1 (2)
C2—C3—H3B	109.5	C26—C25—C24	117.4 (2)
H3A—C3—H3B	108.0	C27—C26—C25	121.6 (3)
N3—C4—C14	124.2 (2)	C27—C26—H26	119.2
N3—C4—H4	117.9	C25—C26—H26	119.2
C14—C4—H4	117.9	C26—C27—C28	122.5 (3)
C19—C18—C23	119.0 (3)	C26—C27—H27	118.7
C19—C18—C17	122.4 (3)	C28—C27—H27	118.7
C23—C18—C17	118.6 (3)		

### Hydrogen-bond geometry (Å, °)

**Table:**

*D*—H···*A*	*D*—H	H···*A*	*D*···*A*	*D*—H···*A*
N2—H2···O2	0.79 (3)	1.99 (3)	2.596 (3)	133 (3)
N3—H3···O1	0.84 (3)	1.88 (3)	2.567 (3)	138 (3)
N3—H3···N1	0.84 (3)	2.62 (3)	2.875 (3)	99 (2)
O5—H5D···O1^i^	0.95 (4)	1.82 (4)	2.747 (3)	164 (5)
N2—H2···O4^ii^	0.79 (3)	2.60 (3)	3.183 (4)	131 (2)
O5—H5C···O2^iii^	0.94 (3)	1.91 (3)	2.839 (3)	170 (3)
C1—H1A···O5^iv^	0.97	2.53	3.409 (4)	151
C2—H2A···O5^iv^	0.97	2.55	3.359 (4)	140

Symmetry codes: (i) −*x*, −*y*, −*z*+2; (ii) −*x*, −*y*, −*z*+1; (iii) −*x*, *y*+1/2, −*z*+3/2; (iv) *x*, −*y*+1/2, *z*−1/2.
